# Flexural failure properties of fiber-reinforced hybrid laminated beam subject to three-point bending

**DOI:** 10.1038/s41598-024-60078-7

**Published:** 2024-04-29

**Authors:** Getahun Tefera, Sarp Adali, Glen Bright

**Affiliations:** Discipline of Mechanical Engineering, University of KwaZulu-Natal, Durban, South Africa

**Keywords:** Flexural failure properties, Carbon/epoxy, Glass/epoxy, Hybrid ratio, Classical lamination plate theory, Engineering, Materials science

## Abstract

The present study investigates the flexural failure properties of a hybrid laminate beam subjected to three-point bending. A symmetrically laminated hybrid beam is constructed using high-strain and inexpensive glass fibre on the top layers and low-strain and expensive carbon fiber on the middle layers. Classical lamination plate theory is used to find the stress and strain distribution that occurs due to the bending moment on the compressive side. The theoretical failure limits of the laminated hybrid beam are analyzed considering the targeted span-to-depth ratios, volume fractions of the fibers and hybrid ratios using the Tsai-Wu failure criterion and Matlab codes. Using the graph of failure index versus hybrid ratios, the minimum thickness of carbon fiber needed for the delay of failure and cost efficiency of the laminated hybrid beam is identified by applying the linear interpolation method. The numerical results indicate that the failure index increases with the increasing loading span and decreases when the volume fraction of fiber increases. In particular, the placement of glass fiber on the top layer of the laminated hybrid beam might have contributed to obtaining higher strains and curvatures before the catastrophic failure properties of carbon fiber. The flexural stiffness of the laminates is found to increase when the hybrid ratio increases. Overall, it is noted that the theoretical analysis is one method that is less time-consuming and cost-effective than other alternative approaches, such as finite element methods and experimental tests to estimate the minimum thickness of high-stiffness and the expensive material needed to maintain the strength and stiffness of the hybrid composite structures over long periods.

## Introduction

Many types of fibers, e.g. carbon, and glass have been used to make fiber-reinforced polymer (FRP) composites using polymers as a matrix material. These FRP materials have several advantages, including lightness, high strength, high stiffness, ease of installation and handling, corrosion resistance, and relatively good durability properties^1–5^. Especially, carbon fiber-reinforced polymer (CFRP), glass fiber-reinforced polymer (GFRP), and a combination of the two materials are most commonly used to develop composite structures applicable to aerospace structures, biomedical components, automobile components, sporting equipment, civil infrastructure, and wind turbine blade structures^6,7^. Carbon fiber has a higher strength-to-weight ratio, stiffness-to-weight ratio, and fatigue resistance compared to glass fiber, but it has a higher cost^8,9^. The relative lower ratio of compressive to tensile strength, a lower strain-to-failure rate, and its inherent catastrophic brittleness properties are disadvantages of CFRP composite material and can hinder their use as structural members that are exposed to flexural and compressive loadings^10^. While GFRP composite material has a higher strain-to-failure ratio, is cheaper, and has a higher weight in comparison to CFRP materials^11,12^. One approach to achieving tailored material properties is to incorporate high-elongation glass and low-elongation carbon fiber embedded in a single polymer matrix material to improve the failure strain^13–16^.

Commonly, glass fiber has been considered a candidate material for hybridization due to its higher strain-to-failure values over carbon fiber. The incorporation of high-modulus carbon fiber into the hybrid composite structures provides higher stiffness and load-bearing capabilities. The low-modulus properties of glass fiber contributed to making the hybrid composite structures more durable and lower the cost of production. Through the hybridization of two types of fiber materials in a polymer matrix, it would be possible to create composite structures that have the advantages of the individual components while diminishing their disadvantages. For example, a high-modulus carbon fiber is required to develop aerospace components, but the catastrophic brittle failure that is usually associated with such a material would be unacceptable. This type of behavior can be optimized by combining different fibers with suitable physical characteristics in a single resin system^17,18^. Additionally, the hybridization of carbon and glass fiber can improve the failure limit and minimize the weight of the composite structures, which can offer an attractive design option for the industry^19–21^. The hybridization process of carbon and glass fibers in a polymer matrix can be achieved by several procedures for different applications. Kalantari et al.^22^ performed multi-objective robust optimization considering minimum weight and cost and subject to a prescribed minimum flexural strength using unidirectional carbon/glass fiber-reinforced hybrid composites under flexural loading. The laminates were constructed with carbon fiber on the tensile side and glass fibre on the compressive side of the composite beam. The result emphasized the importance of hybridization in improving flexural strength and optimizing weight and costs. The presence of glass fibers on the compressive side of the laminated hybrid beam might have contributed to reducing the stress concentration and catastrophic failure properties of carbon fiber, as noted in^23,24^. Dong and Davies^25^ studied the flexural and tensile moduli of S-2 glass and T700S carbon fiber-reinforced hybrid epoxy composites in intra-ply configurations using a three-point bending test under various span-to-depth ratios. The results indicated that flexural modulus increased when the span-to-depth ratio increased from 16 to 32 and became stable as the span-to-depth ratio further increased. Additionally, a change in flexural and tensile moduli was observed when the hybrid ratio varied. Moreover, the flexural properties of bidirectional hybrid epoxy composite specimens reinforced by E-glass and T700S carbon fibers in inter-ply configurations were studied by the same authors^26,26^, which exhibited lowered flexural strength when the partial layers of carbon fiber were replaced by glass fibers.

The effect of stacking sequence, fiber volume fractions, and hybrid ratio may affect the flexural performance of pure and hybrid composite structures. For example, Dong and Davies^27^ identified the effect of stacking sequences on the flexural behavior of carbon and glass fiber-reinforced hybrid laminates. The results confirmed that a positive hybrid effect was observed when the glass fiber-reinforced laminae were placed on the compressive face. While negative hybrid effects were dominant as the glass fiber-reinforced laminae were placed on the tensile face, respectively. An optimal stacking configuration was able to show a positive hybrid effect on the flexural performance of unidirectional and bidirectional hybrid composites, as noted in^28–30^. Khatri and Koczak^31^ used unidirectional and [0/90] fiber orientations to study the flexural response of hybrid composites. The hybrid composite consists of a symmetrically arranged E-glass fiber on the exterior and AS4 graphite in the middle sections, with polyphenylene sulfide (PPS) as matrix material. As expected, the rigidity of the hybrid laminate increased rapidly with increasing AS4/PPS contents. The weak compressive properties of the composite always led to the E-glass fibers delaying the failure initiation in the AS4/PPS layers. Additionally, they observed the importance of a hybrid effect on improving the transverse support and crack-arresting characteristics of the E-glass fibers.

Intralayer hybrid structures were formed for consideration of the dislocation arrangement of fibers in various layers. It reflects the difference in dispersion degrees in the composite. Wang et al.^32^ studied the flexural progressive failure modes of carbon fiber and glass fiber in the interlayer and intralayer configurations of hybrid composites. Results showed that the flexural failure modes for interlayer hybrid composites depend on stacking sequences of carbon and glass fibers. The presence of carbon fiber on the top layer decreases the compressive strain, and the fiber fails early. The flexural failure was delayed when carbon fiber was arranged in the middle or bottom layer. The content of carbon fiber and glass fiber in the intralayer configurations of hybrid composites had an impact on the bending forces. Early failure was observed under increased content of carbon fiber, and the bending force shows a multi-stage fluctuating decline due to the different failure properties of carbon and glass fiber. The delay in bending failure occurred due to increasing the content of glass fiber and exhibited a sharp decline in the bend forces. The layer structures are the critical parameters that affect the flexural performance of the hybrid composites^33^.

Moreover, the influence of hybridization on the tensile and buckling failure properties of hybrid composites was investigated by different authors^34–37^. The tensile failure properties of the fibers depending on the amount of stress concentration that occurs during testing. Fibers having higher stiffness and higher shear properties in the upper layers increased the buckling strength of the hybrid composite samples. Yang et al.^38^ evaluated different failure criteria to develop a new progressive model for predicting the damage evolution of laminated composites subjected to three-point bending. Mainly, damage evolution in the hybrid materials can occur due to delamination. Nowadays, wind turbine blade structures and robot arm components have been produced using hybrid FRP composite material and have been exposed to bending loads during their service lifetimes^39–41^. It is important to do further study to assess the failure properties of those materials.

In this study, theoretical analysis is considered to determine the flexural failure limit of the laminated hybrid composite beam under a three-point bending. Classical laminate plate theory (CLPT) and the Tsai-Wu failure criterion were utilized, assuming that the fibers are perfectly bonded and each layer is homogenous. The hybrid composite laminates were constructed by using T-300 carbon fibre in the middle layer and E-glass fibers in the top and bottom layers. The effect of fiber volume fractions, namely, 45%, 55%, and 65%, were chosen for each fiber type to determine their impact on the delay of failure and cost efficiency. Four span-to-depth ratios of 16, 32, 48, and 64 were implemented in the analytical analysis to assess the failure limits under flexural load. Using the graph of failure index versus hybrid ratios, the minimum amount of carbon fibers needed for the delay of failure on the hybrid composite laminates was estimated. The cost-effective design of the hybrid composite laminate was obtained by using the minimum amount of high-modulus fiber for a given flexural load.

### Laminated hybrid composite beam

In this analytical study, laminated hybrid composite beams reinforced with T-300 carbon fibre and E-glass fiberes are considered to determine the flexural failure limit under a three-point bending^42^. The laminates are preferred to be arranged at ${[{0}_{g}^{0}/0}_{c}^{0}/{0}_{g}^{0}]$ to obtain a better strength from the fibers under bending^43^. Three fiber volume fractions of carbon fiber (${V}_{fc}$) and glass fibers (${V}_{fg}$), such as 45%, 55%, and 65%, are considered in the targeted stacking sequence to find the optimal thickness of carbon fiber needed for delay of failure under different loading spans. For each fiber volume fraction combination, nine stacking configurations were studied at four loading spans, such as 16, 32, 48, and 64. The thickness of the laminated hybrid composite beam is considered to be 4.6 mm thick, with 20 laminas of equal thickness, i.e., the thickness of each lamina is 0.23 mm.

**Figure 1:**
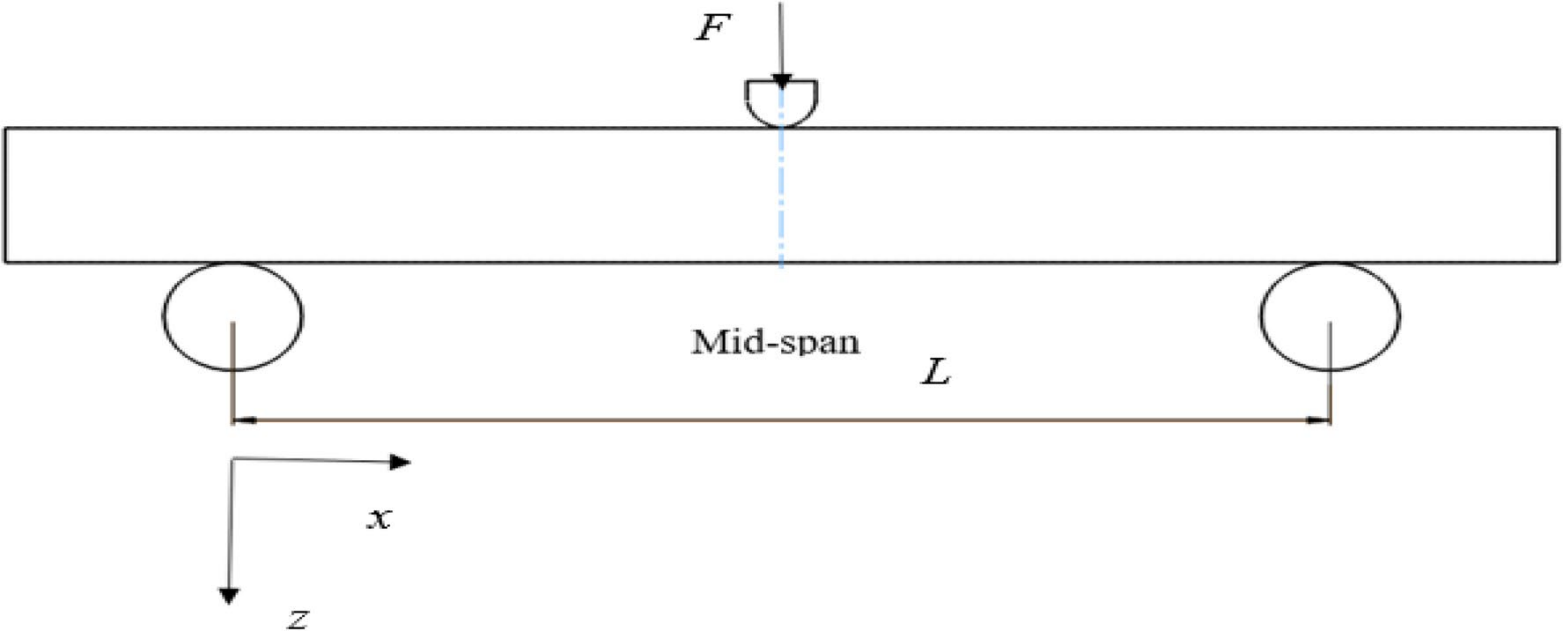
Hybrid laminate subject to three-point bending and the coordinate axes $x$ and $z$.

**Figure 2:**
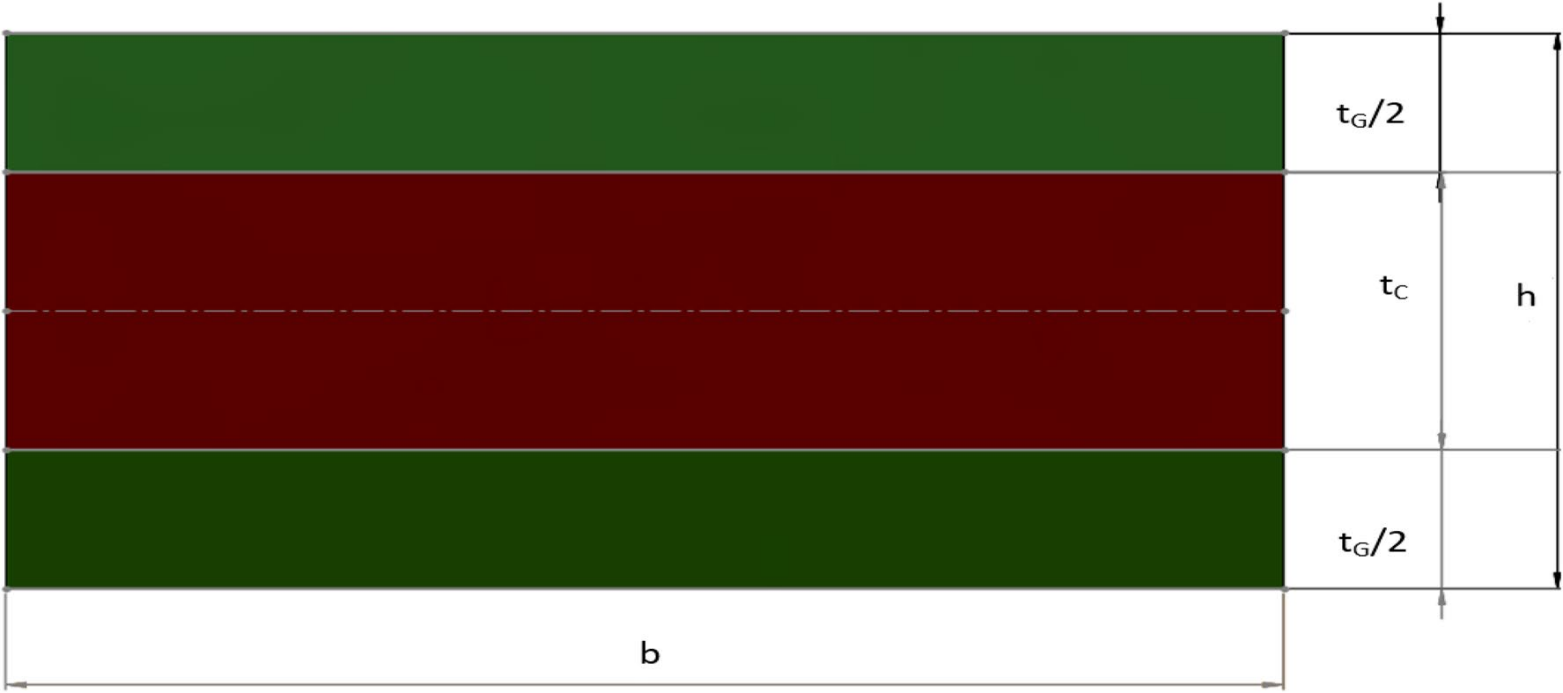
Cross-section of the hybrid composite laminated beam.

A force of 400 N acting on the simply supported laminated hybrid beam is considered for the current analytical study, as shown in Fig. 1. The length (L) and thickness (h) of the beam are represented along $x$ and $z$ axes. Figure 2 shows the cross-sections of the beam having width $b$ and thickness of the hybrid composite beam $h$. The arrangement of a hybrid composite laminate consists of three layers with the top and bottom layers subject to higher strains of glass fiber and the middle layer to lower strain carbon fiber. The top and bottom layers are indicated by the subscript G and the middle layer by the subscript C. The total thickness of the top and bottom layers is ${t}_{G}$ and the thickness of the middle layer is ${t}_{C}$. The total thickness of the hybrid beam laminate (h) is given by:

1 $${\text{h}}={{\text{t}}}_{{\text{G}}}+{{\text{t}}}_{{\text{C}}}$$

The values of the thickness, width and length of the beams are shown in Table 1 for different span-to-depth ($L/h$) ratios. The laminated hybrid composite beam is simply supported and the load $F$ acts at mid-span as shown in Fig. 1. The mechanical properties of the fibers and the matrix materials considered for this study are shown in Table 2.

**Table 1:** Dimensions of the composite laminated beam.

Span-to-depth	Thickness (mm)	Width (mm)	Loading span (mm)
16	4.60	13	73.60
32	4.60	13	147.20
48	4.60	13	220.80
64	4.60	13	294.40

**Table 2:** Mechanical properties of the unidirectional T-300 carbon fiber, E-glass fiber and polymer matrix^44^.

Properties	Unit	Glass	Carbon	Epoxy
Density	kg/m^3^	2600	1750	1200
Axial modulus	GPa	74	230	4.5
Axial shear modulus	GPa	35.42	22	1.308
Axial Poisson’s ratio	–	0.25	0.3	0.4
Axial tensile strength	MPa	1550	2067	72
Axial compressive strength	MPa	1550	1999	102
Transverse tensile strength	MPa	1550	77	72
Transverse compressive strength	MPa	1550	42	102
In-plane shear strength	MPa	35	36	34

The elastic properties of the carbon and glass fiber-reinforced composite, that is, the longitudinal modulus, the transverse modulus, the major Poisson’s ratio, and the in-plane shear modulus, were calculated by using the applicable micromechanical relations given by:

2 $${E}_{11}={E}_{F}{V}_{F}+{E}_{M}{V}_{M}$$

where ${E}_{11}$ is the elastic modulus along the longitudinal directions, ${V}_{F}$ is the volume fraction of the fiber, ${V}_{M}$ is the volume fraction of the polymer matrix, ${E}_{F}$ is the modulus of the fiber, and ${E}_{M}$ is the modulus of the polymer matrix, respectively.

3 $$\frac{1}{{E}_{22}}=\frac{{V}_{F}}{{E}_{F}}+\frac{{V}_{M}}{{E}_{M}}$$

where ${E}_{22}$ is the elastic modulus along transverse directions.

4 $$\frac{1}{{G}_{12}}=\frac{{V}_{F}}{{G}_{F}}+\frac{{V}_{M}}{{G}_{M}}$$

where ${G}_{12}$ is the shear modulus, ${G}_{F}$ and ${G}_{M}$ are the shear modulus of the fiber and polymer matrix^45^. respectively.

5 $${v}_{12}={v}_{F}{V}_{F}+{v}_{M}{V}_{M}$$

where ${v}_{12}$ is the major Poisson’s ratio, ${v}_{F}$ and ${v}_{M}$ are the Poisson’s ratios of the fiber and the polymer matrix.

The ultimate longitudinal and transverse tensile strengths of the composite were calculated based on the failure of the fibers and the polymer matrix. The ultimate transverse compressive strength of the composite lamina was estimated based on the failure of the polymer matrix^39^. The ultimate longitudinal compressive strength of the lamina was estimated based on fiber micro-buckling in the kink mode in compression based on the Lo-Chim model^46^. The longitudinal compressive strength $({X}_{1}^{c})$ and transverse compressive strength (${X}_{2}^{C}$) is given by:

6 $$({X}_{1}^{c})=\frac{{G}_{12}}{1.5+12(6/\pi {)}^{2}\left(\frac{{G}_{12}}{{E}_{11}}\right)}$$

7 $$\left({X}_{2}^{C}\right)={E}_{22}{\varepsilon }_{2}^{C}$$

where ${\upvarepsilon }_{2}^{{\text{C}}}$ is the transverse compressive strain.

The longitudinal tensile strength (${X}_{1}^{T}$) and transverse tensile strength (${X}_{2}^{T}$) properties are denoted by:

8 $${(X}_{1}^{T})={\sigma }_{f}{V}_{f}+{\varepsilon }_{f}{E}_{M}(1-{V}_{f})$$

9 $$\left({X}_{2}^{T}\right)={E}_{22}{\varepsilon }_{2}^{T}$$

where ${\sigma }_{f}$ and ${\varepsilon }_{2}^{T}$ are the maximum strength of the fiber and strain of the polymer matrix. The in-plane shear strength is calculated by:

10 $${S}_{12}^{2}={G}_{12}{\gamma }_{12}$$

where ${\upgamma }_{12}$ is the shear strain of the polymer matrix material.

Mainly, the compressive stress occurs in the top layers of the laminates under the three-point bending. The maximum bending moment at the mid-span of the beam can be obtained by:

11 $$M=\frac{{F}_{max}L}{4b}$$

where ${F}_{max}$ is the applied load.

The strain across the thickness is calculated based on the classical laminated plate theory^30^ and the strains are given by:

12 $$\varepsilon =\left\{\begin{array}{c}{\varepsilon }_{x}\\ {\varepsilon }_{y}\\ {\gamma }_{xy}\end{array}\right\}={\varepsilon }^{0}+zk$$

where ${\upvarepsilon }^{0}$ is the strain vector at the mid-plane and ${\text{k}}$ is the curvature vector of the mid-plane.

The strains under three-point bending are zero at the mid-plane and reach their maximum value at the top and bottom surfaces. The constitutive relation for the laminated beam subject to the normal force $N$ and the bending moment $M$ is given by:

13 $$\left\{\begin{array}{c}N\\ M\end{array}\right\}=\left[\begin{array}{cc}A& B\\ B& D\end{array}\right]\left\{\begin{array}{c}{\varepsilon }^{o}\\ k\end{array}\right\}$$

where $A$ is the extensional stiffness matrix, $B$ is the coupling stiffness matrix and $D$ is the bending stiffness matrix. For a beam subject to three-point bending, the normal force is zero and only the bending moment exists as such Eq. (13) becomes

14 $$\left\{\begin{array}{c}0\\ M\end{array}\right\}=\left[\begin{array}{cc}A& B\\ B& D\end{array}\right]\left\{\begin{array}{c}{\varepsilon }^{o}\\ k\end{array}\right\}$$

In the present case, ${\text{B}}$ matrix is zero and the moment ${\text{M}}$ is given by $$[{\text{M}}]={\left[\begin{array}{ccc}{{\text{M}}}_{{\text{xx}}}& 0& 0\end{array}\right]}^{{\text{T}}}.$$ The curvature $[{\text{k}}]$ is given by:

15 $$\left[k\right]={\left[D\right]}^{-1}[M]$$

The strain on the ${i}^{th}$ lamina is given by ${\varepsilon }_{i}={\varepsilon }^{0}+{z}_{i}k.$ The corresponding stress is ${\sigma }_{i}={Q}_{i}{\varepsilon }_{i}.$ The flexural modulus is calculated based on the equation in^28^:

16 $${F}_{Exx}=\frac{12}{{h}^{3}{D}_{11}^{*}}$$

where ${D}_{11}^{*}$ is the first element of the inverse matrix of $\left[D\right]$.

### Design of the laminated hybrid composite beam

Carbon fiber-reinforced polymers have a low compressive-to-tensile strength and a low strain-to-failure ratio. This limits the application of CFRP material subject to compressive and flexural loads. To improve the mechanical performance and cost-effectiveness of the fibers, a hybrid material that is low-cost and has a high strain-to-failure ratio can be developed^47–50^. Mainly, the presence of higher-stiffness fiber on the top layers of the hybrid laminates contributes to maximizing the stiffness of the composite structures. It is important to find the failure index and the minimum thickness of the costly fibers required to delay the failure of laminated hybrid composites by considering high-strain fibers on the compressive sides of the hybrid laminates. In this design, the failure index $FI$ has to satisfy the inequality $FI\le 1$ for a safe design. In the present case of a hybrid composite laminated beam subject to a three-point loading, the failure point is at the mid-span of the laminated hybrid beam and on the compressive side.

Let ${\alpha }_{ch}$ represent the ratio of the thickness of the carbon fiber reinforced lamina to the total thickness $h$ of the laminated hybrid composite, that is,

17 $${\alpha }_{ch}=\frac{{t}_{C}}{h}$$

where the hybrid ratio ${\alpha }_{ch}=1$ corresponds to carbon fiber-only reinforced laminate and ${\alpha }_{ch}=0$ corresponds to glass fibre-only reinforced laminate. The design objective is to determine the required thicknesses of the high-strain layers (top and bottom glass fiber reinforced layers) and the middle layer (carbon fiber reinforced layer) subject to thickness and failure constraints. The required thickness problem can be expressed as:

18 $$min\,{ \alpha }_{ch}$$

19 $$with\;0\le {\alpha }_{ch}\le 1$$

20 $$subject\;to\;FI\left({\alpha }_{ch}\right)\le 1$$

For a given thickness $h$ and load. The laminate can be composed of glass fibers only if $FI(0)\le 1$. The minimization problem yields a feasible solution for $FI\left(1\right)\le 1$ and a feasible solution does not exist if $FI\left(1\right)>1$. By plotting the graphs of the failure index against the required thickness ratio ${\alpha }_{ch}$, non-failure and failure regions were determined on the hybrid composite laminated beams. Based on the linear interpolation of the graphs for the failure limit $(FL=1)$, the required thickness of carbon fiber layers was computed for optimization of the non-failure limits of the laminated hybrid composite beam.

### Failure prediction

Stress and strain values are required to implement the failure criteria for the FRP composite materials. Presently, several theories exist to predict the failure properties of composite laminates. The Tsai-Wu failure theory is the most commonly used to predicate the failure limit of FRP materials. The Tsai-Wu failure theory is used in the current theoretical analysis, and the numerical simulations were conducted using Matlab programming to compute the values of the stresses and strains. The failure index of the laminates was obtained by considering the maximum load as 400 N. Different volume fractions of the two fibers and span-to-depth ratios were considered at the targeted loading until the first ply failure occurred. Each numerical simulation was compared with the Tsai-Wu failure criterion $(FI\le 1)$ to find the non-failure limit of the laminated hybrid composite beam under the bending load. The maximum bending moment at the mid-span of the laminate, for which $FI>1,$ indicated failure. In this case, failure of the composite material is assumed to occur and a feasible design does not exist for $FI>1.$ The failure index for the Tsai-Wu criterion is defined as:

21 $$FI={F}_{1}{\sigma }_{1}+{F}_{2}{\sigma }_{2}+{F}_{6}{\tau }_{12}+{F}_{11}{\sigma }_{1}^{2}+{F}_{22}{\sigma }_{2}^{2}+{F}_{66}{\tau }_{12}^{2}+2{F}_{12}{\sigma }_{1}{\sigma }_{2}$$

where ${\sigma }_{1}$ the longitudinal stress, ${\sigma }_{2}$ the transverse stress and ${\tau }_{12}$ the in-plane shear stress. The parameters ${F}_{1}$, ${F}_{2}$, ${F}_{6}$, ${F}_{11}$, ${F}_{22}$, ${F}_{66}$ and ${F}_{12}$ are determined using the strength values of the specific composite material (GFRP in the surface layers and CFRP in the middle layer) of the laminate.

$${{F}_{1}=\frac{1}{{X}_{1}^{T}}+\frac{1}{{X}_{1}^{C}} , F}_{2}=\frac{1}{{X}_{2}^{T}}+\frac{1}{{X}_{2}^{C}} , {F}_{11}=-\frac{1}{{X}_{1}^{T}{X}_{1}^{C}}$$

$${F}_{22}=-\frac{1}{{X}_{2}^{T}{X}_{2}^{C}} , {F}_{12}=-\frac{1}{2}\sqrt{{F}_{11}{F}_{22}} {F}_{66}=\frac{1}{{S}_{12}^{2}}$$

## Numerical results and discussion

The dimensions of the laminated hybrid composite beam and the mechanical properties of carbon fiber, glass fiber, and polymer matrix are represented in Tables 1 and 2. The mechanical properties of composite lamina were obtained using micromechanical analysis, considering the material properties represented in Table 2. The arrangement of the layers of the laminated hybrid composite was considered to be ${[{0}_{g}^{0}/0}_{c}^{0}/{0}_{g}^{0}]$, to obtain the optimum bending strength. The subscripts g and c stand for GFRP and CFRP composite materials. In this study, glass fiber is considered on the top and bottom layers of the laminates to find its high strain property on the flexural performance of hybrid laminates. Three fiber volumes fractions such as 45%, 55%, and 65% were used to develop the hybrid laminates beam using the two fibers. The theoretical flexural failure and mechanical properties of the laminated hybrid beam at different hybrid ratios and span-to-depth ratios were investigated using the Tsai Wu failure criteria, considering the First Ply Failure (FPF) method. The fibers are symmetrically aligned along the $x$ direction and the bending moment, ${M}_{xx}$, being applied, as shown in Fig. 1. For the present work, 400 N was considered to determine the failure limits of composite laminate beams under flexural loading.

### Failure indices

**Figure 3:**
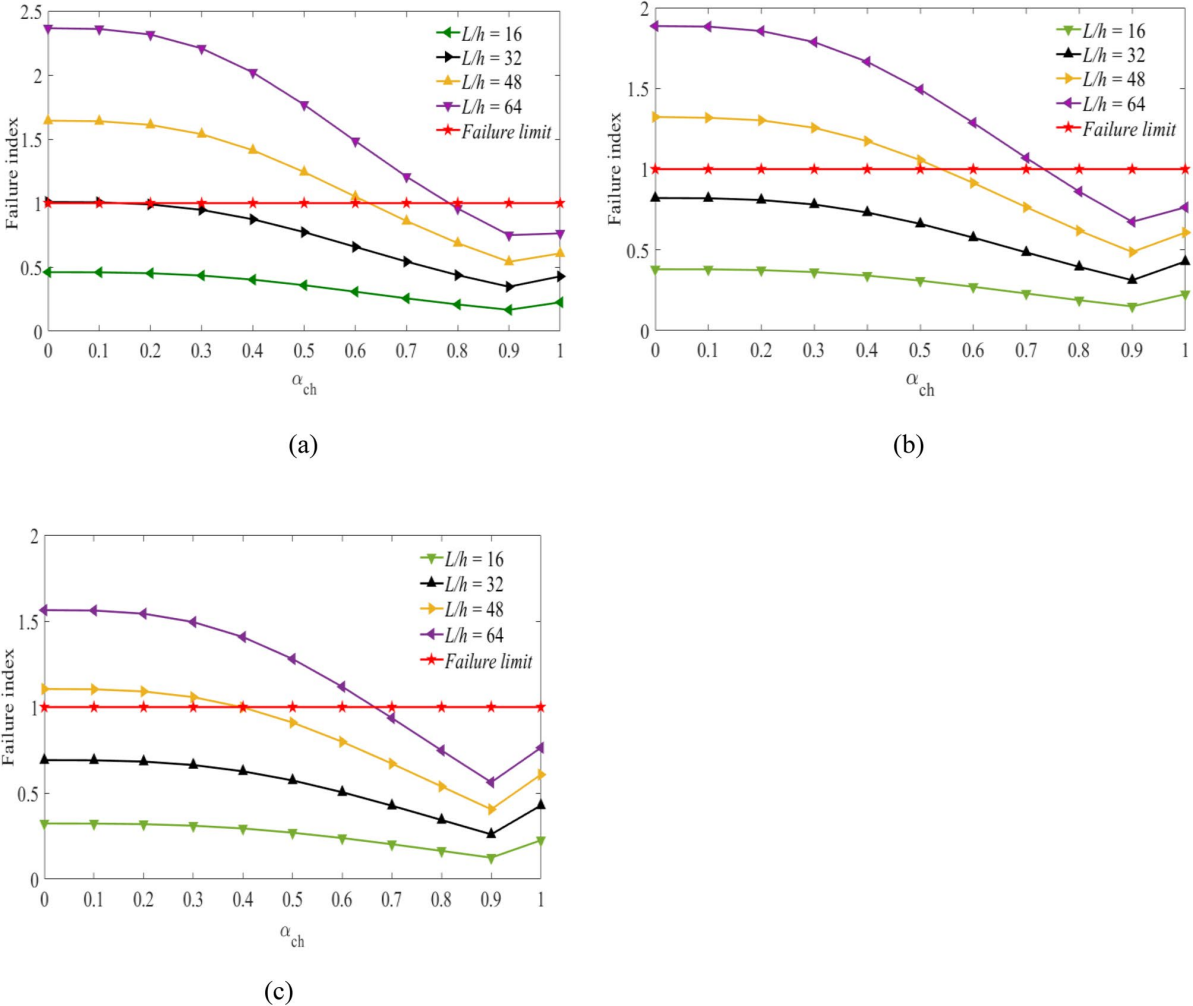
Failure index vs hybrid ratio for laminated beam $\left[ {0_{g}^{0} /0_{c}^{0} /0_{g}^{0} } \right]$ with ${V}_{fc}=45\%$ and for (**a**) ${V}_{fg}=45\%$, (**b**), ${V}_{fg}=55\%$, (**c**) ${V}_{fg}=65\%$ for the targeted span-to-depth ratios.

**Figure 4:**
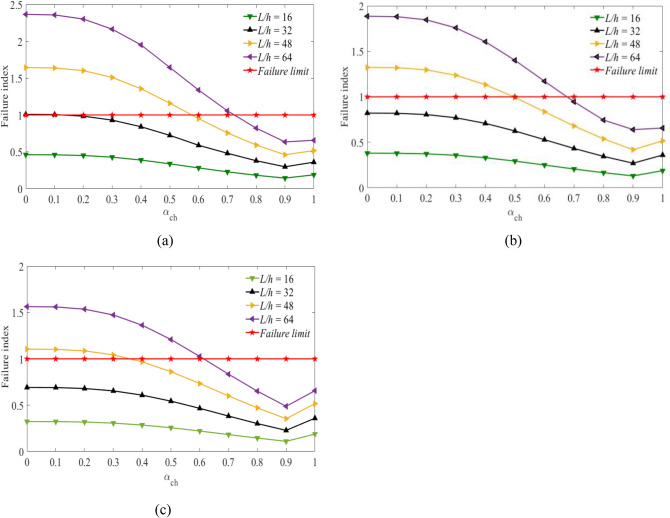
Failure index vs hybrid ratio for laminated beam $[{0}_{g}^{0}/{0}_{c}^{0}/{0}_{g}^{0}]$ with ${V}_{fc}=55\%$ and for (**a**) ${V}_{fg}=45\%$, (**b**), ${V}_{fg}=55\%$, (**c**) ${V}_{fg}=65\%$ for the targeted span-to-depth ratios.

**Figure 5:**
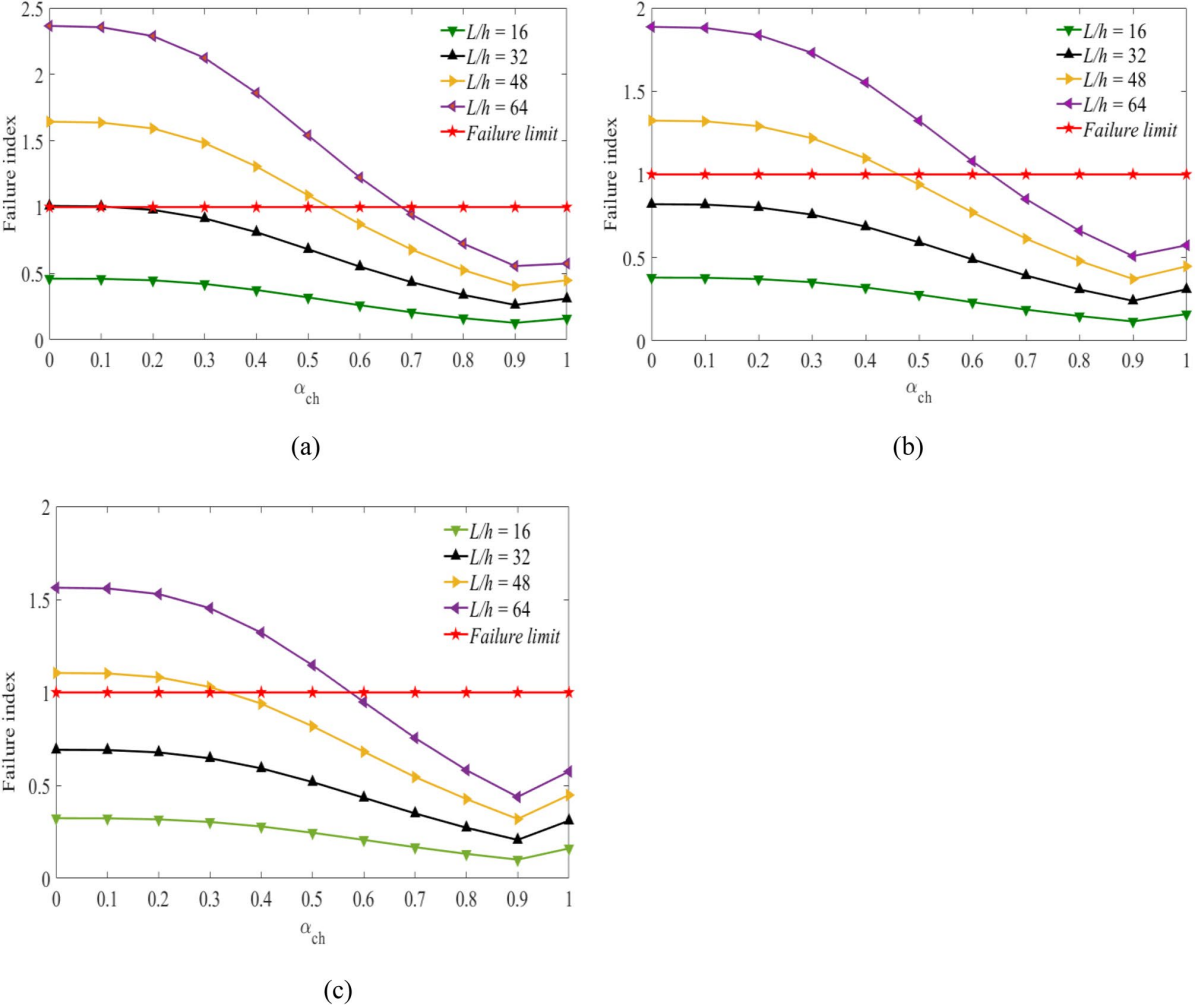
Failure index vs hybrid ratio for laminated beam $[{0}_{g}^{0}/{0}_{c}^{0}/{0}_{g}^{0}]$ with ${V}_{fc}=65\%$ and for (**a**) ${V}_{fg}=45\%$, (**b**), ${V}_{fg}=55\%$, (**c**) ${V}_{fg}=65\%$ for the targeted span-to-depth ratios.

Figures 3, 4 and 5 show the values of failure indices obtained at a stacking sequence $[{0}_{g}^{0}/{0}_{c}^{0}/{0}_{g}^{0}]$ of the hybrid laminates as the fiber volume fractions of carbon fiber are considered to be ${V}_{fc}=45\mathrm{\%}$, ${V}_{fc}=55\mathrm{\%}$ and ${V}_{fc}=65\mathrm{\%}$. The *x*-axis represents the values of the hybrid ratio (${\alpha }_{ch}={t}_{C}/h)$ for $0\le {\alpha }_{ch}\le 1$. When the values of ${\alpha }_{ch}=0$, corresponds to a laminates beam constructed using pure glass fiber. While the values of ${\alpha }_{ch}=1$, the laminated beam was developed using pure carbon fiber.

In Fig. 3, the values of the failure index are obtained considering the volume fraction of carbon fiber ${V}_{fc}=45\mathrm{\%}$. As the volume fraction of carbon fiber is considered to be ${V}_{fc}=55\mathrm{\%}$ and ${V}_{fc}=65\mathrm{\%}$, the results are shown in Figs. 4 and 5 with the span-to-depth ratios of $L/h=16, 32, 48,\mathrm{ and }\;64$ to a point load of $F=400N$. It can be seen from Fig. 3(a) that the minimum hybrid ratios needed for non-failure of the hybrid beam $FI\le 1$ are obtained as the values of ${\alpha }_{ch}=0.16, 0.63, {\text{and}}\;0.78$. These values are obtained when the volume fraction of glass fiber is considered to be ${V}_{fg}=45\%$ at span-to-depth ratios of 32, 48, and 64, respectively. The critical values of the hybrid ratios depend on the volume fraction of glass fiber. As observed in Fig. 3(b), the minimum hybrid ratios needed for non-failure of the beam laminates are changed to ${\alpha }_{ch}=0.54\;{\text{and}}\;0.73$ at span-to-depth ratios of 48 and 64. The change in results is obtained when the volume fraction of the glass fiber increases to 55%. As the volume fraction of glass fiber increases to ${V}_{fg}=65\%$ as shown in Fig. 3(c), the minimum thicknesses of carbon fiber needed for non-failure of the hybrid beams are reduced to ${\alpha }_{ch}=0.40\;{\text{and}}\;0.67$ during the span-to-depth ratios of 48 and 64, respectively. In the case of a span-to-depth ratio of 16, the failure index is less than one in all cases, and no laminated hybrid composite beam failure occurs. The thickness of the carbon fiber and glass fiber in the hybrid laminates can be determined based on the values of the hybrid ratio. When the hybrid ratio is 1, the laminated beam is produced with pure carbon fiber. As shown in Fig. 3, increasing the hybrid ratio results in a decrease in the failure index and delays the failure of the laminates. This occurred due to increasing the thickness of carbon fiber and decreasing the thickness of glass fiber in the hybrid laminates. Carbon fibre has a lower strain and higher mechanical properties compared to glass fiber. Meanwhile, as the hybrid ratio approaches 1, a slight increment is observed, and no failure is observed. This might occur due to the lower strain properties of the carbon fiber, which increase the stress in the laminates. The curves below the failure limits are safe from failure. The curves above the failure limits need more layers of carbon fiber than glass fiber in the hybrid laminates to be safe from failure. Increasing the volume fraction of the fibers has a direct effect on the delay of the failure properties of the hybrid laminates. The theoretical results confirmed the importance of carbon fiber for the delay of failure in the hybrid laminated composite beam. When the thickness of carbon fiber in the hybrid laminates decreases, it is necessary to increase the volume fraction of glass fiber to delay the failure of the hybrid beam laminates.

Figure 4(a,b,c) shows the values of the failure index against the hybrid ratio for $[{0}_{g}^{0}/{0}_{c}^{0}/{0}_{g}^{0}]$ of hybrid beam laminates. In this case, the volume fraction of carbon fiber is considered to be ${V}_{fc}=55\%$. While the volume fraction of glass fibers is assumed to be ${V}_{fg}=45\%, 55\%, and\;65\%$ on the specified span-to-depth ratios. The thickness of the two fibers needed for the delay in the failure of the hybrid laminated beam can be obtained from the curves as shown in Fig. 4(a). Non-failure of the laminated beam is observed when the values of ${\alpha }_{ch}=0.14, 0.58\;and\;0.72$ during the span-to-depth ratios of 32, 48, and 64. This happened due to increasing the thickness of carbon fiber in the hybrid laminates. The hybrid ratios are decreased by 14.3%, 8.6%, and 8.3% compared to the results obtained in Fig. 3(a). Additionally, the values of the hybrid ratio are obtained from the curves at ${V}_{fg}=55\%$ as shown in Fig. 4(b). No beam failure is observed during the span-to-depth ratios of 16 and 32. It occurred due to the lower loading span of the hybrid beam, which has a higher strength. As the span-to-depth ratios increase to 48 and 64, failure has occurred in the laminated beams. The minimum values of hybrid ratios needed for non-failure of the laminated beams are ${\alpha }_{ch}=0.50\;and\;0.67$, respectively. Moreover, the amount of carbon and glass fiber needed for the delay in the failure of the hybrid laminated beams is assessed in Fig. 4(c). In this case, the volume fraction of glass fiber was considered to be ${V}_{fg}=65\%.$ The amounts of hybrid ratio (thickness of carbon and glass fiber in the laminates) needed to delay the failure of the laminated beams are ${\alpha }_{ch}=0.36\;and\;0.61$ during the span-to-depth ratios of 48 and 64. In this case, the hybrid ratios are decreased by 11.1% and 9.8% compared to the case shown in Fig. 3(c). It is possible to reduce the middle layer's thickness by increasing the volume fraction of carbon fibers to delay the failure of the hybrid laminated beam, as expected. In other words, increasing the volume fraction of carbon fiber in the hybrid laminates leads to a decrease in the hybrid ratios, which increases the lifetime.

The minimum thickness of carbon fibers needed to be safe from failure was obtained from the graph of failure index versus hybrid ratio, as shown in Fig. 5. The volume fraction of carbon fiber is considered to be ${V}_{fc}=65\%$. As shown in Fig. 5(a), the required hybrid ratios needed for the non-failure of the hybrid beam laminates are ${\alpha }_{ch}=0.15, 0.54\;{\text{and}}\;0.68$ during the span-to-depth ratios of 32, 48, and 64. The hybrid ratios are decreased by about 33.3%, 16.7% and 14.7% during span-to-depth ratios of 32, 48 and 64 compared to the results obtained in Fig. 3(a), respectively. Whereas, the required hybrid ratios needed for the non-failure of the hybrid beams are ${\alpha }_{ch}=0.46\;{\text{and}}\;0.63$ during the span-to-depth ratios of 48 and 64, as observed in Fig. 5(b). In the case of Fig. 5(c), the minimum values of hybrid ratios needed for the non-failure of the hybrid bema laminates are ${\alpha }_{ch}=0.34\;{\text{and}}\;0.57$ during the span-to-depth ratios of 48 and 64, respectively. The failure properties of hybrid laminates under the specified loading spans are assessed using Figs. 3, 4, and 5. The results show that the failure of the hybrid laminates increased when the loading span increased. These properties are minimized by increasing the volume fractions of the fibers and increasing the thickness of the carbon fiber.

Based on the results obtained from Figs. 3, 4, and 5, the minimum thickness of carbon fiber needed to avoid failure decreased as the thickness of glass fiber increased in the hybrid laminated beam. The extent of the decrease in the hybrid ratio consequently decreases the material cost and strength, while the lifetime of the hybrid laminates is lowered in the specified span-to-depth ratios.

### Flexural modulus

**Figure 6:**
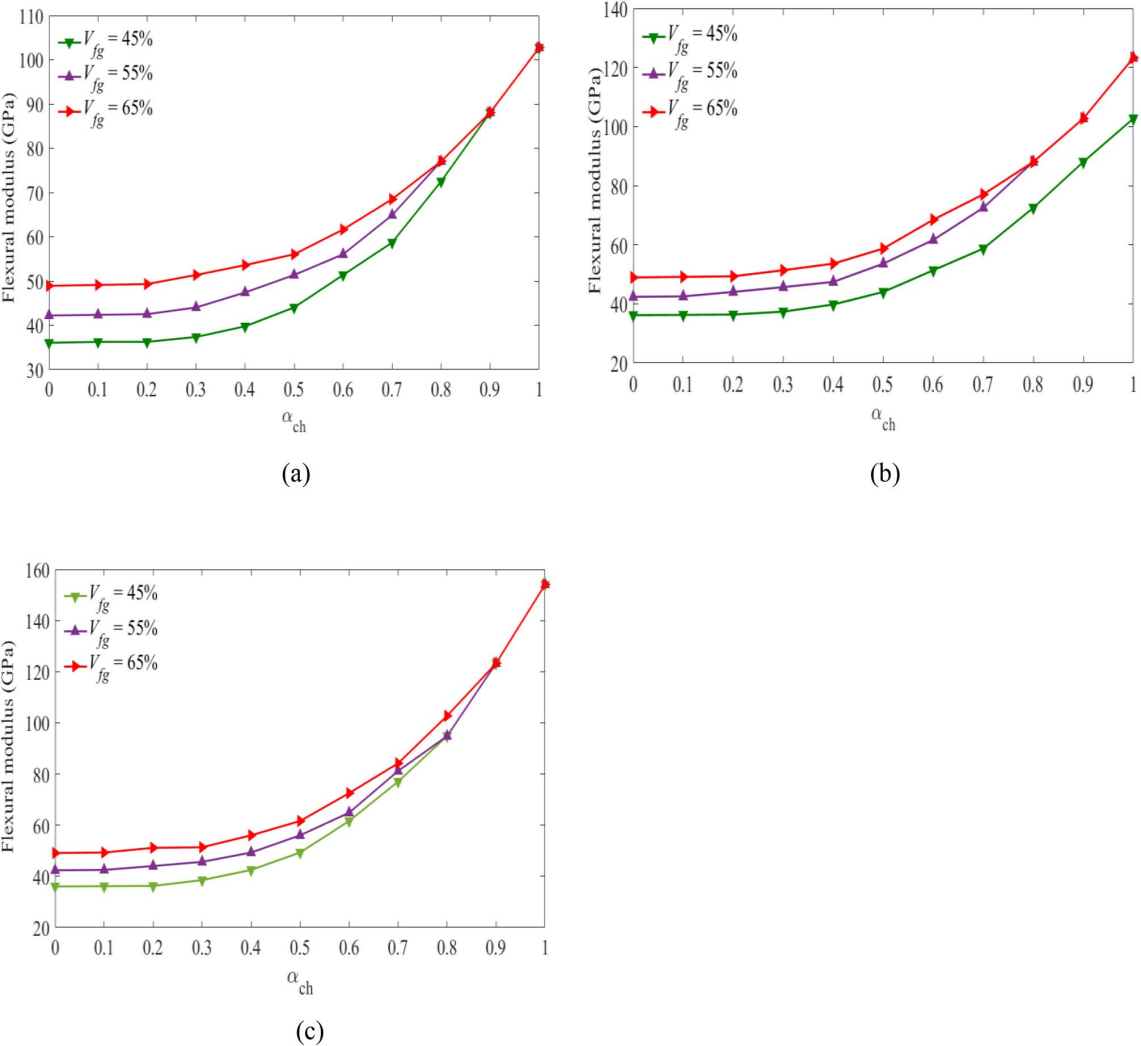
Flexural modulus vs hybrid ratio for laminated beam $[{0}_{g}^{0}/{0}_{c}^{0}/{0}_{g}^{0}]$ for (**a**) ${V}_{fc}=45\%$, (**b**), ${V}_{fc}=55\%$, (**c**) ${V}_{fc}=65\%$ for the specified ${V}_{fg}$ values.

The flexural modulus of the laminated hybrid beams is calculated using Eq. (16). Results presented in Fig. 6(a, b,c) show the values of flexural modulus plotted against the hybrid ratio considering the volume fraction of the middle layers of carbon fiber at ${V}_{fc}=45\%, 55\%, {\text{and}}\;65\%$. As shown from the curves, the flexural modulus increases with an increasing hybrid ratio, as expected. An increase in the flexural modulus is observed and becomes more pronounced for ${\mathrm{\alpha }}_{{\text{ch}}}\ge 0.5$. For ${\mathrm{\alpha }}_{{\text{ch}}}\le 0.4$, the change in the flexural modulus is observed to be fairly minor.

## Maximum stress

**Figure 7:**
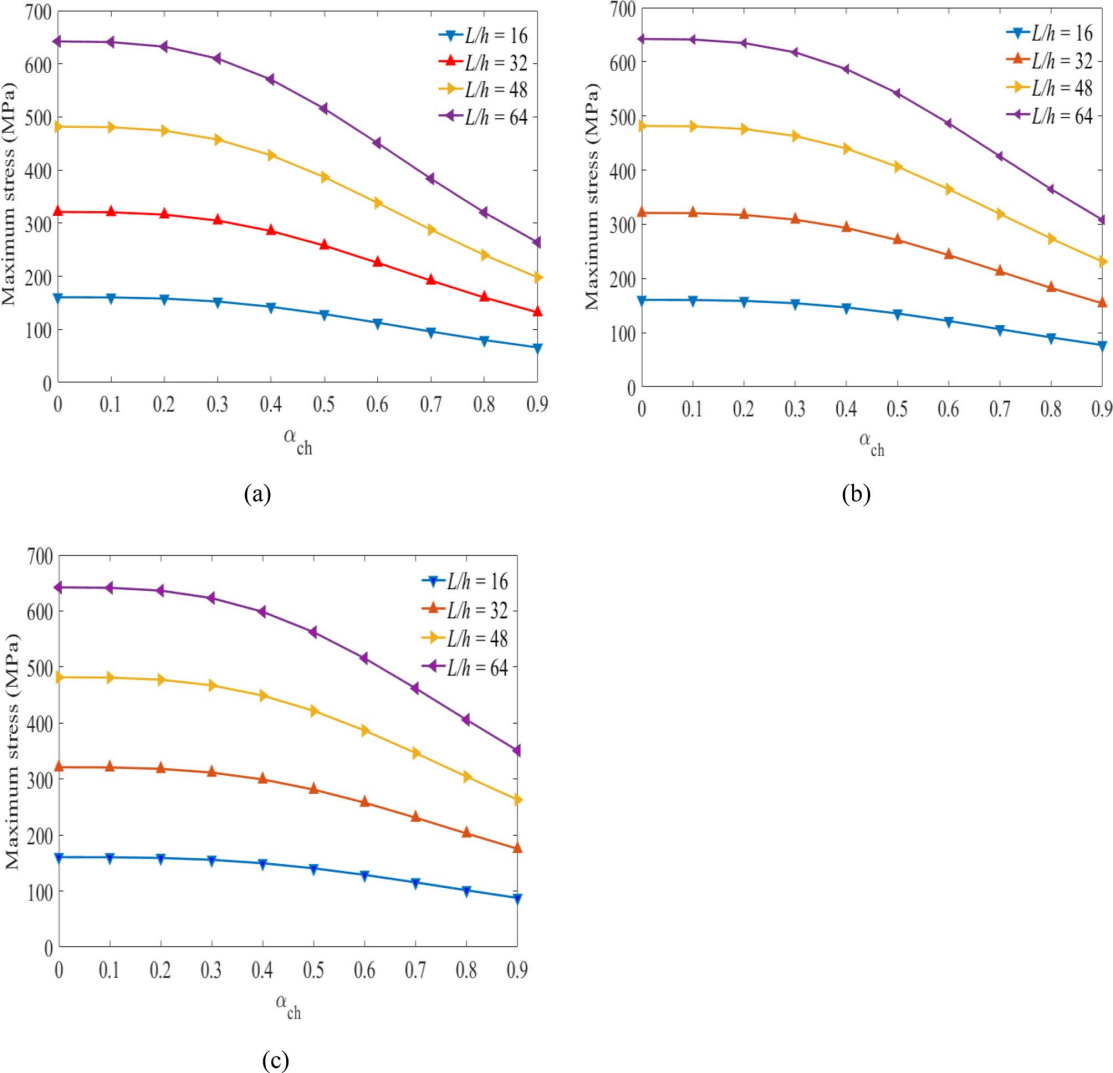
Maximum stress plotted against hybrid ratio for laminated hybrid beam $[{0}_{g}^{0}/{0}_{c}^{0}/{0}_{g}^{0}]$ with ${V}_{fc}=45\%$ and for (**a**) ${V}_{fg}=45\%$, (**b**), ${V}_{fg}=55\%$, (**c**) ${V}_{fg}=65\%$ for the targeted span-to-depth ratios and loading.

**Figure 8:**
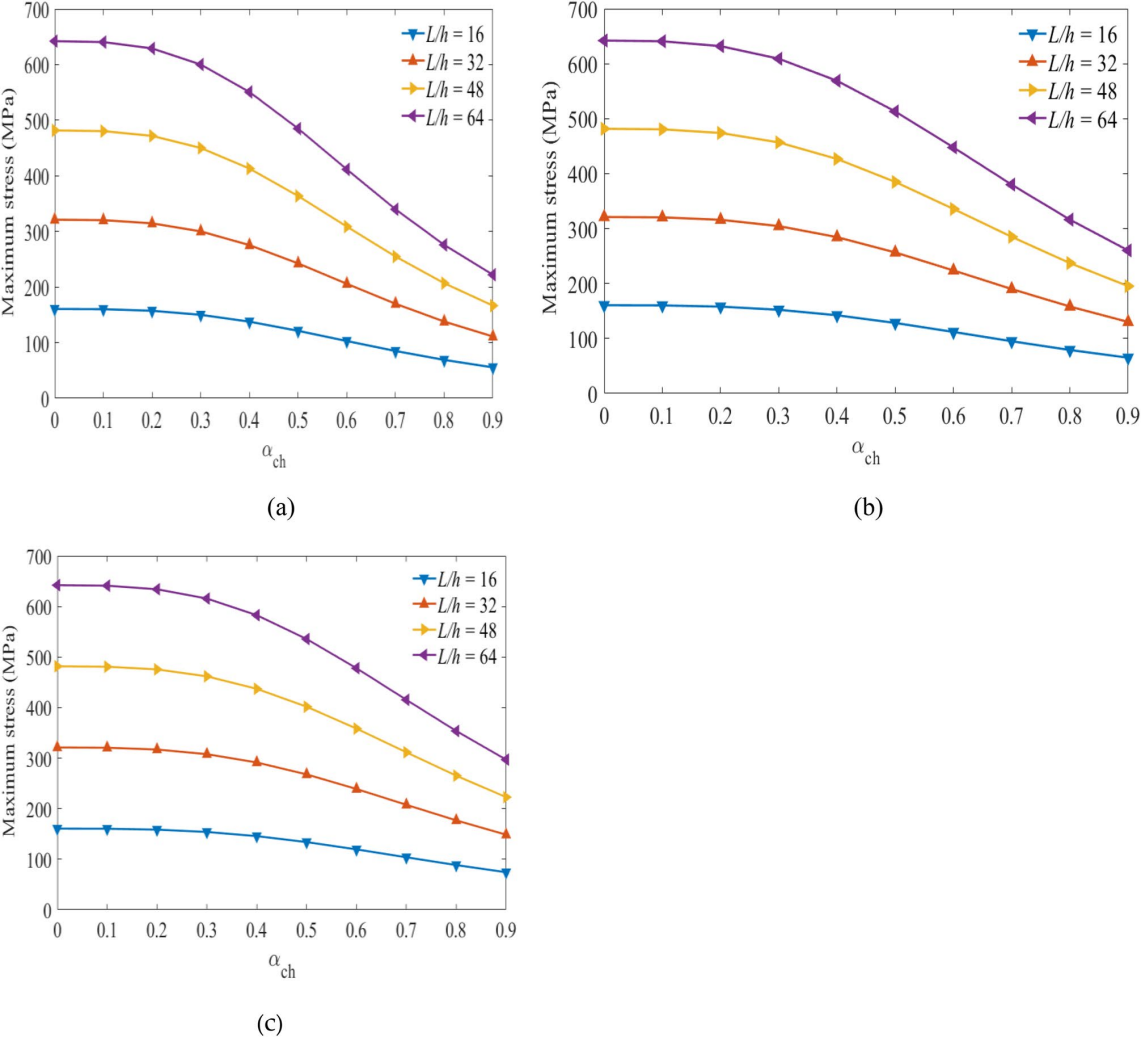
Maximum stress plotted against hybrid ratio for laminated hybrid beam $[{0}_{g}^{0}/{0}_{c}^{0}/{0}_{g}^{0}]$ with ${V}_{fc}=55\%$ and for (**a**) ${V}_{fg}=45\%$, (**b**), ${V}_{fg}=55\%$, (**c**) ${V}_{fg}=65\%$ for the targeted span-to-depth ratios and loading.

**Figure 9:**
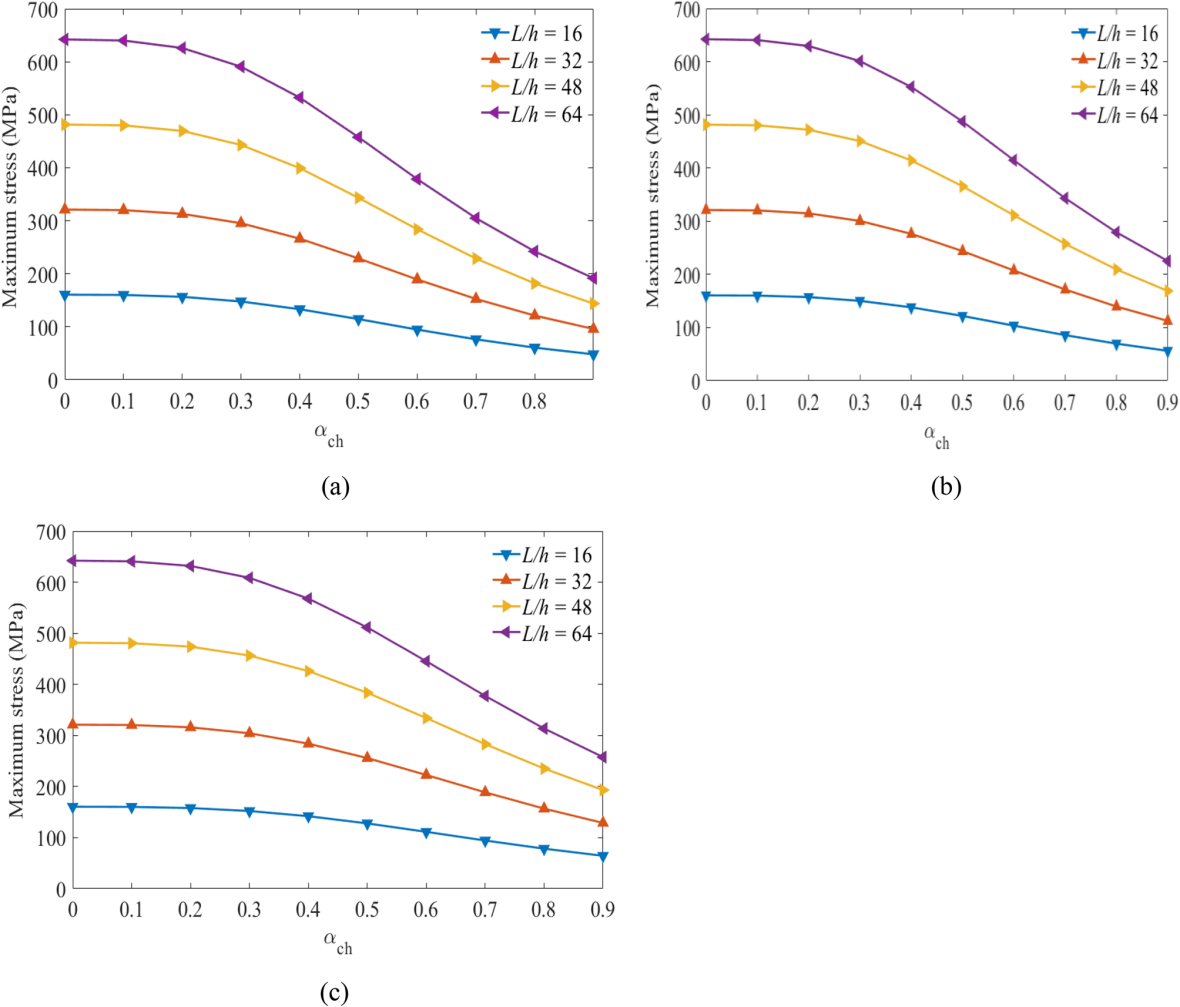
Maximum stress plotted against hybrid ratio for laminated hybrid beam $[{0}_{g}^{0}/{0}_{c}^{0}/{0}_{g}^{0}]$ with ${V}_{fc}=65\%$ and for (**a**) ${V}_{fg}=45\%$, (**b**), ${V}_{fg}=55\%$, (**c**) ${V}_{fg}=65\%$ for the targeted span-to-depth ratios and loading.

Next, the relationships between the stress values versus the hybrid ratios are obtained considering the specified volume fractions of glass and carbon fiber under different span-to-depth ratios and loading. Figures 7, 8, and 9 show the maximum values obtained at the top sections of the laminated hybrid beams. The stacking sequence $[{0}_{g}^{0}/{0}_{c}^{0}/{0}_{g}^{0}]$ are considered to find the maximum stress values between ${0\le \mathrm{\alpha }}_{{\text{ch}}}\le 1$ under the targeted span-to-depth ratios and loadings. Mostly, optimal stress values can be obtained on $[{0}_{g}^{0}/{0}_{c}^{0}/{0}_{g}^{0}]$ stacking sequences compared to other orientations^43^. The volume fraction of carbon fiber in the middle layers of the laminated hybrid beam is considered to be ${V}_{fc}=45\mathrm{\%}$*,* ${V}_{fc}=55\mathrm{\%}$ and ${V}_{fc}=65\mathrm{\%}$. Figures 7(a,b,c) show the values of stresses obtained at the top layer of the laminated hybrid beam at ${{\text{V}}}_{{\text{fc}}}=45\mathrm{\%}$ for the targeted span-to-depth ratios and flexural loading. It is observed that the values of maximum stress obtained are reduced as the thickness of carbon fiber increases, as expected. When the hybrid ratio increases from 0.0 to 0.5, the values of maximum stress decrease due to the presence of more carbon fiber in the laminated hybrid beam. This indicated that the lifetime of the laminated hybrid composite beam is increased due to the presence of high-stiffness carbon fiber on it.

Figures 8(a–c) show the values of maximum stress plotted against the hybrid ratios when ${V}_{fc}=55\%$ at the specific span-to-depth ratios and flexural loading. The values of the maximum stress obtained on the top layer of the laminated hybrid beam decreased by 32.4%, 25.1%, and 19.9%. This occurs when the hybrid ratio increases from 0 to 0.5 and the volume fraction of glass fiber increases from $45\;to\;65\%,$ respectively. Increasing the thickness of the middle layer leads to a decrease in the maximum stress on the top layer of the lamina, and consequently, a delay in the failure of the hybrid beam laminates might happen.

Figures 9(a–c) show the values of maximum stress versus hybrid rations when ${V}_{fc}=65\%$ during different span-to-depth ratios and flexural loading. The theoretical results indicated that the reduction in the stress values at the top layers reaches 40.30%, 31.73%, and 25.56% as the hybrid ratio and volume fraction of the glass fiber content changed from 0 to 0.5, and the volume fraction of glass fibre increases from $45\;to\;65\%,$ respectively. Characterization of the hybrid laminate, under various volume fraction fibers and hybrid ratios, can optimize the compressive stress on the top layers, and support the delay of failure of the material. In all cases, as the span-to-depth ratio increased, the flexural stress on the top layers increased and the lifetime reduced. While the change in flexural stress is minimal as the span-to-depth ratio increases.

## Conclusions

The minimum cost design problem is addressed for laminated hybrid composite beams made of high-strain and inexpensive glass fiber in the surface layers and low-strain and expensive carbon fiber in the middle layers to obtain the advantages of the two fiber materials. Symmetrical stacking sequence $[{0}_{{\text{g}}}^{0}/{0}_{{\text{c}}}^{0}/{0}_{{\text{g}}}^{0}]$ is used to attain improved flexural strength and stiffness properties. Considering the first ply failure (FPF) methods, the stress and strain on the top layers of the laminated hybrid beam are determined using the classical laminate plate theory (CLPT). The theoretical flexural failure limit of laminated hybrid beams is obtained by considering different fiber volume fractions of the two fibers and span-to-depth ratios using the Tsai-Wu failure criterion and Matlab programming codes. The minimum thicknesses of glass and carbon fibre materials required to avoid flexural failure on the laminated hybrid beam are obtained by plotting the failure index values as a function of the hybrid ratios by applying the linear interpolation method. As expected, the failure index increased with increasing span-to-depth and decreased when the volume fraction of the fibers increased. The stiffness of the hybrid composite laminates increases as the hybrid ratio increases. Mainly, the placement of glass fibre on the top layer of the hybrid laminates might contribute to obtaining high strains and curvatures to lower the stress on the lamina and delay failure. Overall, it is noted that the theoretical analysis is the one method that is less time-consuming and cost-effective than other alternative approaches, such as finite element methods and experimental tests to estimate the minimum thickness of high-stiffness and the expensive material needed to maintain the strength and stiffness of the hybrid composite structures for an extended period.

## Data Availability

Data presented in the study are available in the article.
